# Analysis of common bean expressed sequence tags identifies sulfur metabolic pathways active in seed and sulfur-rich proteins highly expressed in the absence of phaseolin and major lectins

**DOI:** 10.1186/1471-2164-12-268

**Published:** 2011-05-26

**Authors:** Fuqiang Yin, Agnieszka Pajak, Ralph Chapman, Andrew Sharpe, Shangzhi Huang, Frédéric Marsolais

**Affiliations:** 1 Department of Bioscience and Biotechnology, School of Life Sciences, Sun Yat-sen University, Guangzhou 510275, China; 2 Agriculture and Agri-Food Canada, Southern Crop Protection and Food Research Centre, 1391 Sandford St., London, ON N5V 4T3 Canada; 3 National Research Council of Canada, Plant Biotechnology Institute, 110 Gymnasium Place, Saskatoon, SK S7N 0W9 Canada; 4 Department of Biology, University of Western Ontario, London, ON N6A 5B7 Canada

## Abstract

**Background:**

A deficiency in phaseolin and phytohemagglutinin is associated with a near doubling of sulfur amino acid content in genetically related lines of common bean (*Phaseolus vulgaris*), particularly cysteine, elevated by 70%, and methionine, elevated by 10%. This mostly takes place at the expense of an abundant non-protein amino acid, *S*-methyl-cysteine. The deficiency in phaseolin and phytohemagglutinin is mainly compensated by increased levels of the 11S globulin legumin and residual lectins. Legumin, albumin-2, defensin and albumin-1 were previously identified as contributing to the increased sulfur amino acid content in the mutant line, on the basis of similarity to proteins from other legumes.

**Results:**

Profiling of free amino acid in developing seeds of the BAT93 reference genotype revealed a biphasic accumulation of gamma-glutamyl-*S*-methyl-cysteine, the main soluble form of *S*-methyl-cysteine, with a lag phase occurring during storage protein accumulation. A collection of 30,147 expressed sequence tags (ESTs) was generated from four developmental stages, corresponding to distinct phases of gamma-glutamyl-*S*-methyl-cysteine accumulation, and covering the transitions to reserve accumulation and dessication. Analysis of gene ontology categories indicated the occurrence of multiple sulfur metabolic pathways, including all enzymatic activities responsible for sulfate assimilation, *de novo *cysteine and methionine biosynthesis. Integration of genomic and proteomic data enabled the identification and isolation of cDNAs coding for legumin, albumin-2, defensin D1 and albumin-1A and -B induced in the absence of phaseolin and phytohemagglutinin. Their deduced amino acid sequences have a higher content of cysteine than methionine, providing an explanation for the preferential increase of cysteine in the mutant line.

**Conclusion:**

The EST collection provides a foundation to further investigate sulfur metabolism and the differential accumulation of sulfur amino acids in seed of common bean. Identification of sulfur-rich proteins whose levels are elevated in seed lacking phaseolin and phytohemagglutinin and sulfur metabolic genes may assist the improvement of protein quality.

## Background

Common bean (*Phaseolus vulgaris*) is the most important leguminous food crop grown for dry seed worldwide, both in acreage and yield. Historically, this species has been an important model for the study of seed storage proteins [1]. In commercial cultivars, the 7S globulin phaseolin constitutes approximately half of total seed protein. Lectins are also abundant, with phytohemagglutinins and α-amylase inhibitors accounting for 10% and 5% of seed protein, respectively. Like in other grain legumes, the content of essential sulfur amino acids is sub-optimal for nutrition. A strategy proposed to improve protein quality and bioavailability of sulfur amino acids consists in the selection and breeding of highly-digestible phaseolin types [2]. A different approach may rely on variation in storage protein composition.

Osborn et al. developed genetically related lines integrating mutations conferring a deficiency in phaseolin and major lectins, which are encoded by unique loci [3]. The arcelin-phytohemagglutinin-α-amylase inhibitor (APA) locus was introgressed from G12882, a wild accession containing arcelin-1, into the commercial cultivar Sanilac (white navy bean), to generate the SARC1 line. Recessive mutations from *Phaseolus coccineus *and 'Great Northern 1140' were introgressed into the SARC1 background, conferring a deficiency in phaseolin and lectins, respectively. SMARC1-PN1 lacks phaseolin and SMARC1N-PN1 lacks phaseolin, phytohemagglutinin and arcelin. SARC1, SMARC1-PN1 and SMARC1N-PN1 share a similar level (ca. 85%) of the recurrent parental Sanilac background. Introgression of the APA locus containing arcelin-1 from wild *P. vulgaris *is associated with resistance to major storage pests, the weevils *Zabrotes subfasciatus *and *Acanthoscelides obtectus *[4-6]. However, in the absence of detailed molecular information about the APA locus, the identity of the lectin(s) conferring this resistance remains elusive [7,8].

The deficiency in phaseolin and major lectins, phytohemagglutinin and arcelin, results in a nearly two-fold increase in sulfur amino acid content in seed, particularly of Cys, elevated by 70%, and Met, by 10% [9]. This takes place mostly at the expense of *S*-methyl-Cys, an abundant non-protein amino acid which cannot substitute for Met or Cys in the diet [10]. Proteomic analysis revealed that the lack of phaseolin and major lectins was mainly compensated by increases in the 11S globulin legumin and residual lectins, particularly the β subunit of α-amylase inhibitor-1, α-amylase inhibitor-like protein, mannose lectin FRIL, and leucoagglutinating phytohemagglutinin encoded by *PDLEC2 *[11]. Several proteins contributing to the increased Cys content including legumin, albumin-2, defensin and albumin-1 were identified on the basis of similarity to related proteins from other legumes. Based on quantification by 2-D gel electrophoresis, legumin levels were raised by 3-fold, to 17% of total protein, while albumin-2 was elevated by 10-fold, to 1.2% of total protein. Defensin and albumin-1 could not be quantified accurately as they were characterized from selective extracts.

A significant number of expressed sequence tags (ESTs) have been generated from root, leaf and pod of common bean [12-17], but a similar resource is lacking for the seed, despite its nutritional importance. In addition, transcript profiling studies of common bean using high density arrays have so far relied on cross-specific hybridization to soybean platforms [18,19], in part due to a lack of relevant sequence information. The objective of this study was to generate a common bean EST collection which would provide a foundation to further investigate the metabolism of sulfur amino acids in developing seeds, by using a functional genomic approach. Four seed developmental stages were sampled in the reference genotype BAT93 [20], corresponding to distinct stages of accumulation of γ-Glu-*S*-methyl-Cys, the major soluble form of *S*-methyl-Cys. *In silico *analysis revealed the occurrence of several pathways of sulfur metabolism ranging from sulfate assimilation to (homo)glutathione biosynthesis. Analysis of EST clusters provided detailed information on the identity and abundance of storage protein transcripts. Integration with proteomic data enabled isolation of cDNAs coding for legumin, albumin-2, defensin and albumin-1 isoforms which are elevated in the absence of phaseolin and major lectins. These proteins have a higher Cys than Met content, providing an explanation for the preferential increase of Cys in SMARC1N-PN1.

## Results

### Selection of seed developmental stages and generation of ESTs

**Figure 1:**
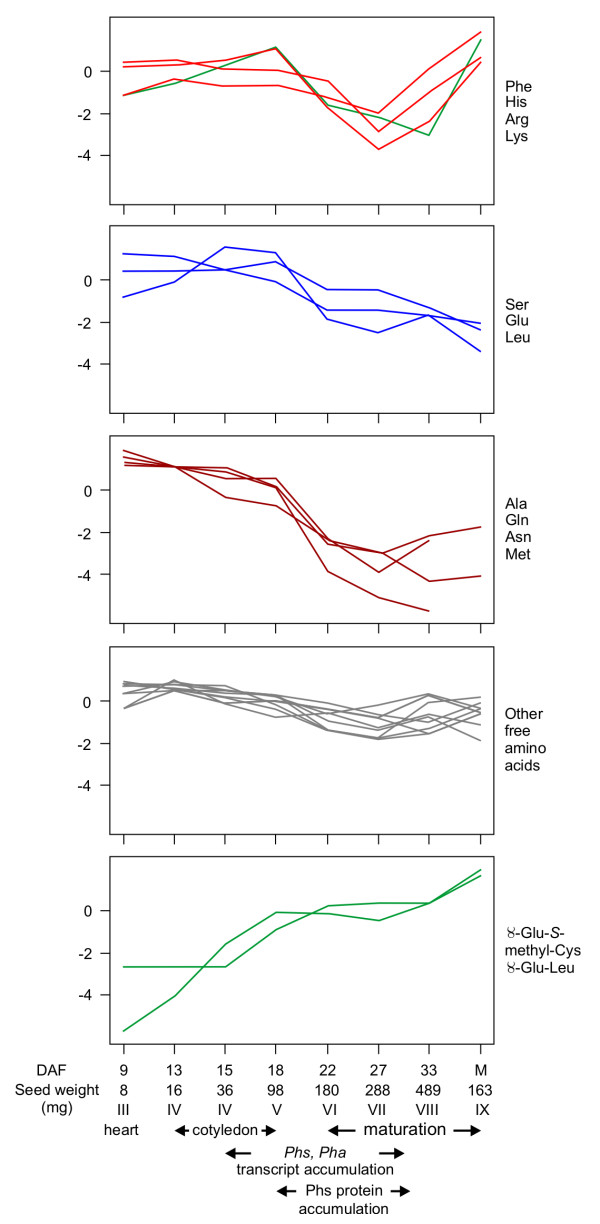
**Cluster analysis of free amino acid profiles during seed development**. Amino acids were classified using *k*-means analysis into five groups. The names of amino acids belonging to each group are indicated on the right. Data on the y axis is the amino acid content (see Additional file 1) normalized to the average and expressed in log_2 _scale. Developmental stages are named according to Walbot et al. [21]. Timing of phaseolin (*Phs*) and phytohemagglutinin (*Pha*) transcript and phaseolin protein accumulation is according to Bobb et al. [22].

Free amino acids were profiled at eight developmental stages of BAT93 seed, to identify stages suitable to generate ESTs (see Additional File 1). Developmental stages are designated after Walbot et al. [21]. According to this nomenclature, storage protein transcripts reach their maximal levels between stages IV (cotyledon) to VI (maturation), while storage products are accumulated between stages V (cotyledon) to VII (maturation) [22]. During stage VIII (maturation) the seed undergoes desiccation. Amino acid content was normalized over the average of developmental stages, expressed in log_2 _scale, and *k*-means analysis performed to reveal common developmental patterns. Amino acids were grouped into five clusters (Figure 1). The levels of most free amino acids decreased markedly between stages V to VII during which protein reserves are actively accumulated. The nitrogen-rich amino acids, Arg, His and Lys formed a cluster with Phe. Amino acids in this cluster were characterized by a marked rise in their levels during the transition to seed desiccation and dormancy. The second and third clusters contained central intermediates in amino acid metabolism, Ser, Glu, Ala, Gln and Asn, along with Leu and Met. Their levels generally decreased throughout seed development. This decline was more pronounced for amino acids in the third cluster which included Met. γ-Glu-*S*-methyl-Cys and γ-Glu-Leu formed a unique cluster as their levels rose throughout seed development. γ-Glu-S-methyl-Cys showed a biphasic curve of accumulation, with a continuous rise until stage V, followed by a plateau until stage VII, and a resumption of accumulation from stage VIII (see Additional file 1, Figure 1). In comparison, the levels of free *S*-methyl-Cys were high at stages III and IV, and decreased at stages V and VI, in parallel with Met levels.

Four developmental stages were selected to generate ESTs, corresponding to distinct phases of γ-Glu-S-methyl-Cys accumulation: stage IV - cotyledon [14 days after fertilization (DAF), 29 mg seed weight] and stage V - cotyledon (16 DAF, 48 mg seed weight), coinciding with early γ-Glu-S-methyl-Cys accumulation, stage VI - maturation (21 DAF, 164 mg seed weight), coinciding with the lag phase, and stage VIII - maturation (30 DAF, 380 mg seed weight), coinciding with the resumption of accumulation. A total of 8,725, 9,537, 5,260 and 6,625 ESTs were generated for each respective developmental stage. Of these, Gene Ontology (GO) annotation from *Arabidopsis *[23] was retrieved for 5,511, 3,825, 1,561 and 3,303 ESTs, respectively. Assembly of the total 30,147 ESTs yielded 3,658 contigs and 6,027 singletons.

### In silico analysis of ESTs identifies features of sulfur and amino acid metabolism in developing seeds

**Figure 2:**
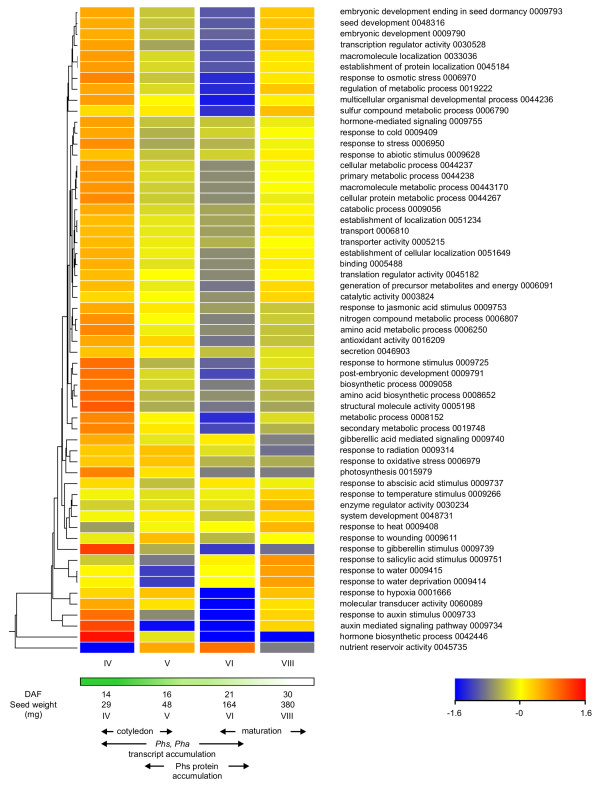
**Cluster analysis of general GO category profiles during seed development**. GO categories were classified by hierarchical clustering analysis. The percentage of ESTs assigned to a GO category (see Additional file 2) was normalized to the average and expressed in a log_2 _scale. GO category name and number is shown on the right. The horizontal bar represents seed color related to photosynthetic capacity [21].

**Figure 3:**
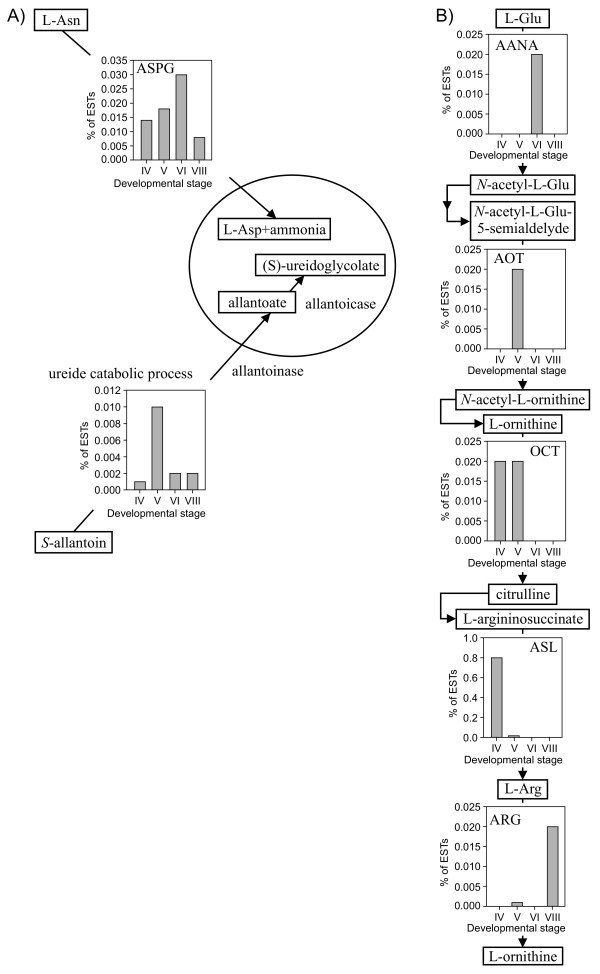
**Representation of GO categories related to nitrogen supply (A) and Arg metabolic pathway (B) during seed development**. Percentage of ESTs is displayed for each developmental stage. Abbreviations and GO category numbers are as follows: ASPG: asparaginase activity - 0004067; ureide catabolic process - 0010136 (allantoinase and allantoicase); AANA: amino acid *N*-acetyltransferase activity - 0004042 (*N*-acetyl Glu synthase); AOT: acetylornithine transaminase activity - 0003992; OCT: carboxyl or carbamoyltransferase activity - 0016743 (ornithine carbamoyltransferase); ASL: argininosuccinate lyase activity - 0004056; ARG: arginase activity - 0004053.

The representation of general GO categories of biological process and molecular function was analyzed during seed development. The percentage of ESTs assigned to a GO category (see Additional file 2) was normalized to the average over seed development, expressed in log_2 _scale and submitted to hierarchical clustering analysis (Figure 2). Categories of cellular metabolism, primary metabolism, macromolecule metabolism and cellular protein metabolism clustered together. Their representation suggested that general metabolic activity was highest at stage IV, progressively decreased until stage VI and then increased at stage VIII. The same general trend was observed for the categories of amino acid and sulfur compound metabolic processes. The decline in metabolic activity coincided with accumulation of storage products. Indeed, the nutrient reservoir activity category was most highly represented at stage VI, followed by stage V. The latter clustered away from all other categories. Hormone biosynthesic process also had a unique profile, being highest at the first stage of development and markedly down at the last two stages. The percentage of ESTs assigned to the category of photosynthesis was highest at stage IV, followed by stage V, and further decreased at stages VI and VIII, consistent with the loss of chlorophyll pigmentation in cotyledons during seed maturation [21]. Interestingly, categories of photosynthesis, response to oxidative stress and response to radiation were grouped together. The category of response to water deprivation had highest levels at stage VIII, corresponding to seed desiccation. Analysis of GO categories related to the supply of nitrogen to developing seeds indicated that ureide catabolism, comprising allantoinase and allantoicase activities, was most highly represented at stage V, coinciding with the onset of storage protein accumulation, whereas the percentage of ESTs associated with asparaginase activity increased steadily until stage VI, the mid-point of storage protein accumulation (Figure 3A).

**Figure 4:**
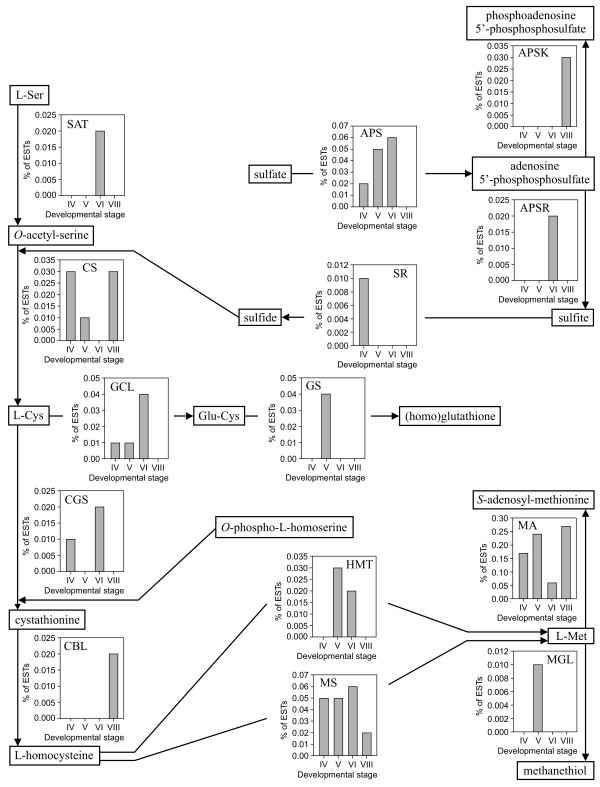
**Representation of GO categories related to sulfur amino acid metabolic pathways during seed development**. Percentage of ESTs is displayed for each developmental stage. Abbreviations and GO category numbers are as follows: APS: sulfate adenylyltransferase activity - 0004781; APSK: kinase activity - 0016301 (adenylyl-sulfate kinase); APSR: adenylyl-sulfate reductase activity - 0009973; CBL: cystathionine β-lyase activity - 0004121; CGS: cystathionine γ-synthase activity - 0003962; CS: Cys synthase activity - 0004124; GCL: Glu-Cys ligase activity - 0004357; GS: (homo)glutathione synthase activity - 0004363; HMT: homocysteine *S*-methyltransferase activity - 0008898; MA: Met adenosyltransferase activity - 0004478; MGL: Met γ-lyase activity - 0018826; MS: Met synthase activity - 0008705; SAT: Ser acetyltransferase activity - 0004781; SR: sulfite reductase activity - 0016002.

Five different GO categories were associated with Arg metabolism (Figure 3B). Of these, argininosuccinate lyase activity, the last step in Arg biosynthesis, was represented by a high percentage of ESTs at stage IV, of 0.80%. This correlates with an increase in steady-stage Arg levels between stages III to V (see Additional File 1). Analysis of categories related to sulfur metabolism indicated that all enzymatic activities associated with sulfur assimilation, *de novo *Cys and Met biosynthesis were represented in the EST dataset (Figure 4). These include sulfate adenylyltransferase, adenylyl-sulfate reductase and sulfite reductase; Ser *O*-acetyltransferase and Cys synthase; and cystathionine γ-synthase, cystathionine β-lyase and Met synthase. Homocysteine *S*-methyltransferase, involved in the production of Met via the *S*-methylmethionine cycle, the catabolic enzyme Met γ-lyase and the two enzymes involved in (homo)glutathione biosynthesis, Glu-Cys ligase and (homo)glutathione synthase, were also represented.

### Analysis of seed protein ESTs

Clustering of ESTs is an efficient tool to estimate the identity and abundance of transcripts in a complex mRNA sample. This analysis focused on the sulfur-poor 7S globulin phaseolin and lectins, the most abundant seed proteins in commercial cultivars [1], and on the sulfur-rich 11S globulins, 2S albumins, and defensins whose levels are elevated in the absence of phaseolin and phytohemagglutinin [11]. To identify contigs coding for sulfur-rich proteins induced in mature seed of SMARC1N-PN1, conceptual translations of sequences were compared with *de novo *sequenced peptides from previously obtained liquid chromatography-tandem mass spectrometry (LC-MS/MS) data [11]. Corresponding cDNAs were isolated.

#### Sulfur-poor 7S globulin phaseolin and lectins

**Figure 5:**
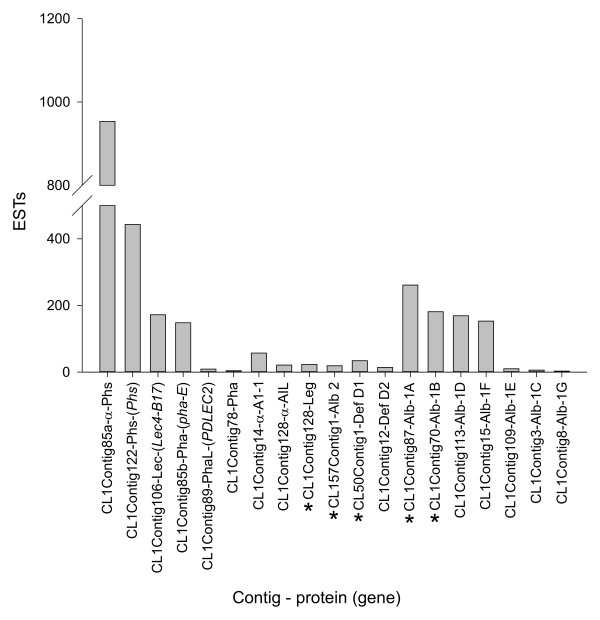
**Transcript expression of seed proteins in the BAT93 line**. Phaseolin and lectin contigs were annotated on the basis of nearest BLASTx hit in the UniProt database, with e-value ranging between 0 to 1.00E-115. For sulfur-rich proteins, only full-length contigs are represented. Contigs coding for sulfur-rich proteins elevated in SMARC1N-PN1, lacking phaseolin, phytohemagglutinin and arcelin, are marked with an asterisk. Phs, phaseolin; Lec, lectin; Pha, phytohemagglutinin; PhaL, leucoagglutinating phytohemagglutinin; AI, amylase inhibitor; AIL, amylase inhibitor-like protein.

Within this subset, most ESTs were derived from the genes encoding the α- and β-subunit of S-type phaseolin, which differ by the presence of a 27 base pair insertion, and are present in cultivated varieties representative of the Mesoamerican gene pool [24,25] (Figure 5). The lectin encoded by *lec4-B17 *and phytohemagglutinin encoded by *pha-E *[26] were the next most highly represented, followed by α-amylase inhibitor-1 [27] and α-amylase inhibitor-like protein [28]. Fewer ESTs were observed for the leucoagglutinating phytohemagglutinin encoded by *PDLEC2 *[29], and an additional phytohemagglutinin previously isolated from an arcelin-5 genotype [30]. The phytohemagglutinin encoded by *pha-L *[26] and mannose lectin FRIL [31], previously identified in SARC1 and SMARC1N-PN1 [11], were not represented in the BAT93 EST dataset. Conversely, the arcelin-5 phytohemagglutinin was not previously identified in SARC1 or SMARC1N-PN1 [11]. There was no evidence for additional phaseolin or lectin transcripts in BAT93. The phytohemagglutinins identified here are encoded by distinct genes, as they share only between 80 to 87% identity in their deduced amino acid sequence.

#### Sulfur-rich 11S globulins, 2S albumins and defensins

Among sulfur-rich proteins, clustering of ESTs yielded three legumin, three albumin-2, four defensin and eleven albumin-1 contigs or singletons. Within each protein type, the isoform induced in SMARC1N-PN1 was encoded by the most highly represented contig in the BAT93 EST collection (Figure 5).

**Figure 6:**
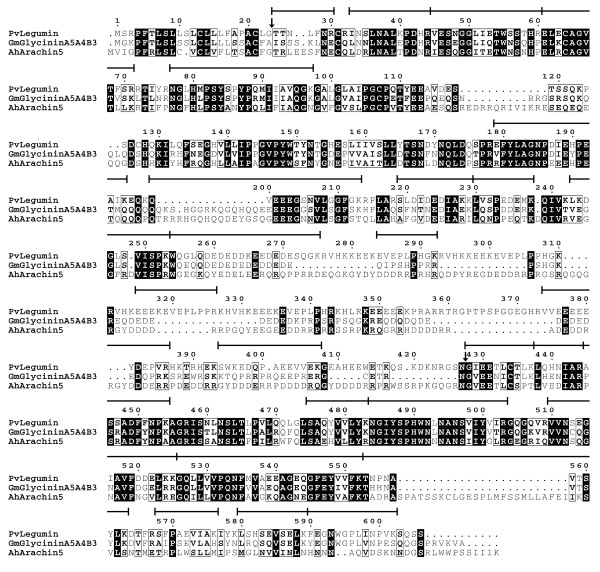
**Alignment of deduced amino acid sequences of *P. vulgaris *legumin and closely related 11S globulins**. Vertical arrows mark cleavage sites after the signal peptide and between the α- and β-subunits. Horizontal bars indicate *de novo *sequenced tryptic peptides from 2-D gel spots (see Additional file 3A) [11]. Species codes are as follows: Pv, *P. vulgaris*; Gm, *G. max*; and Ah, *A. hypogaea*.

**Figure 7:**
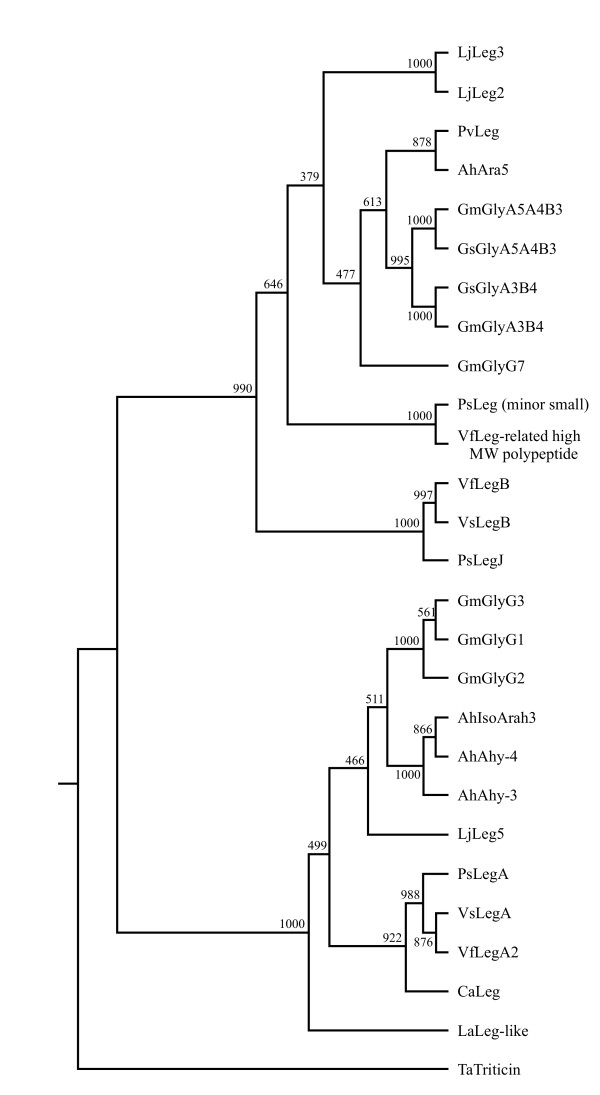
**Phylogenetic tree of 11S globulins**. Numbers at the fork of the unrooted tree indicate the number of times the group of sequences to the right of that fork occurred among a data set of 1,000 trees. Abbreviations are as follows: Leg, legumin; Ara, arachin; and Gly, glycinin. Species codes are as in Figure 6 and also include: Lj, *L. japonicus*; La, *Lupinus albus*; Gs, *Glycine soja*; Vf, *V. faba*; Vs, *Vicia sativa*; Ps, *P. sativum*; Ca, *C. arietinum*; and Ta, *Triticum arvense*.

The major legumin cDNA encodes a protein of 606 amino acids (Figure 6). Removal of the signal peptide, spanning residues 1 to 23, generates a precursor with a predicted molecular weight of 66.3 kD. Cleavage after the conserved Asn^427 ^residue gives rise to an acidic α-subunit, with a predicted molecular weight of 46.4 kD and pI of 5.45, and a β-subunit, with a predicted molecular weight of 19.8 kD and pI of 7.03. The α-subunit contains a Glu-rich domain, spanning positions 260 to 415. Conventional N-terminal and *de novo *peptide sequencing support the location of the cleavage sites [11,32]. *De novo *sequenced tryptic peptides from replicate samples of 2-D gel spots [11] covered 48 and 79% of the deduced sequences of the α- and β-subunits, respectively (Figure 6 and see Additional file 3A). The *P. vulgaris *legumin amino acid sequence shares 54 and 41% identity with glycinin A5A4B3 from *Glycine max *and arachin 5 from *Arachis hypogaea *[33], respectively, but is more similar in length to arachin 5. Phylogenetic analysis indicated that the *P. vulgaris *protein belongs to a group of 11S globulins from legumes, comprising B-type, Met-poor legumins from *Vicia *species [34] (Figure 7). The *P. vulgaris *legumin is part of a subgroup of high molecular weight legumins which includes group-2 glycinins from soybean [35], arachin 5, legumin storage proteins 2 and -3 from *Lotus japonicus *[36], the minor small legumin from *Pisum sativum *[37] and legumin-related high molecular weight polypeptide from *Vicia faba *[38]. Members of this subgroup are characterized by an extended C-terminal half of the α-subunit arising by expansion and mutation of a sequence repeat.

**Figure 8:**
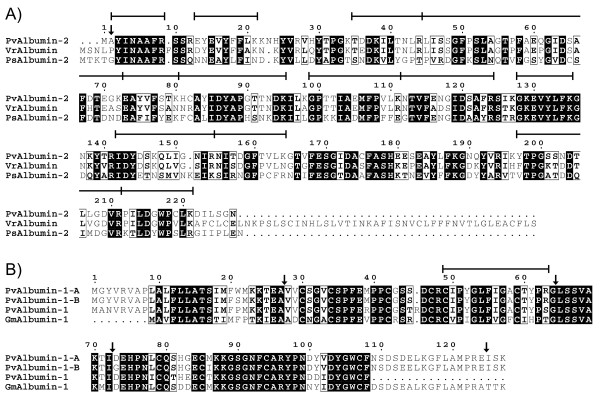
**Alignment of deduced amino acid sequences of *P. vulgaris *albumin-2 (A) and albumin-1 (B) with related proteins**. Vertical arrows mark cleavage sites of polypeptide precursors. Horizontal bars indicate *de novo *sequenced tryptic peptides from 2-D gel spots and SDS-PAGE bands from methanol soluble extracts (see Additional file 3B) [11]. Species codes are as in Figures 6 and 7 and also includes: Vr, *V. radiata*.

The albumin-2 cDNA encodes a protein of 277 amino acids with 80% identity to a seed albumin from *Vigna radiata *and 49% identity to albumin-2 from *P. sativum *[39] (Figure 8A). The protein contains four hemopexin-like repeats implicated in polyamine binding [40]. *De novo *sequenced tryptic peptides from replicate samples of 2-D gel spots covered 74% of the deduced sequence of the mature polypeptide (Figure 8A and see Additional file 3B) [11]. The *de novo *sequencing data indicated that the N-terminal Met residue is absent in the mature polypeptide.

The defensin highly expressed in SMARC1N-PN1 had been previously identified on the basis of its similarity to a defensin from *Cicer arietinum *[11]. However, associated tryptic peptide sequences actually belong to antifungal defensin D1 from *P. vulgaris *[41], whose partial cDNA and N-terminal amino acid sequences were absent from databases. The defensin D1 cDNA isolated here encodes a precursor of 75 residues with 93% identity to defensin D2 from *V. radiata *[42]. The mature peptide is characterized by a Cys-stabilized αβ motif, consisting of three β-strands and one α-helix held by four disulfide bridges. The two tryptic peptides previously identified [11], spanning positions 30 to 39 and 40 to 53, are the only ones within range of detection by the mass spectrometer. The second most abundant contig, and associated defensin D2 cDNA, encode a precursor polypeptide with 93% identity to a defensin from *Vigna unguiculata*. Defensins D1 and -2 are 37% identical in amino acid sequence.

Two albumin-1 cDNAs, albumin-1A and -B may encode the methanol-soluble albumin-1 highly expressed in SMARC1N-PN1. Their deduced amino acid sequences are 97% identical. They encode polypeptide precursors of 127 amino acids with 88% identity to a partial albumin-1 from *P. vulgaris *[43], and 71% identity to albumin-1 from *G. max *[44] (Figure 8B). The precursors give rise to chains b (residues 28 to 64) and a (residues 73 to 124) [45]. The b chain is arranged in a knottin-fold containing three β-strands held by three disulfide bridges [46,47]. The peptide previously *de novo *sequenced spans residues 49 to 63 of the b chain [11]. The presence of an Arg at position 63 generates a tryptic site unique to albumin-1A and -B, among the albumin-1 isoforms represented in the EST dataset.

Availability of cloned cDNAs provides information about the sulfur amino acid content of sulfur-rich proteins whose levels are elevated in SMARC1N-PN1. The mature legumin subunits contain 0.9% of their residues as Cys and 0.7% as Met. Mature albumin-2 contains 1.3% of Cys and 0.4% of Met. Mature defensin D1 is particularly rich in Cys at 17% and lacks Met residues. The mature albumin-1A and -B subunits have a Cys content of 11%, and a Met content of 3.3 and 2.2%, respectively.

### Purification of legumin

**Figure 9:**
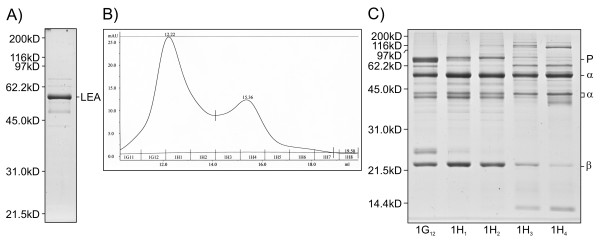
**Purification of abundant proteins of 56 kD from SMARC1N-PN1 seeds**. SDS-PAGE of purified group 3 late embryogenesis abundant protein **(A)**, and legumin fractions **(C) **with their elution profile **(B)**. LEA: group 3 late embryogenesis abundant protein; P, α, and β refer to prolegumin and its subunits.

To further characterize legumin, this protein was purified from SMARC1N-PN1 by targeting the most abundant 2-D gel spots, 78 to 80 (59-64 kD and pI value of 5.5-5.6), corresponding to its α-subunit and representing 10% of total protein [11]. The apparent pI value was used to select conditions for purification by ion exchange chromatography. Fractions were screened by SDS-PAGE for bands having the appropriate molecular weight. Ammonium sulfate precipitation followed by ion exchange chromatography yielded two fractions of interest, characterized by size exclusion chromatography. Proteins were identified after in-gel trypsin digestion and LC-MS. The first fraction contained group 3 late embryogenesis abundant protein with an apparent molecular weight of 56.0 kD (Figure 9A; see Additional file 3C). In 2-D gels, this protein migrated to spots 84 (64 kD and pI value of 5.4) and 104 (55 kD and pI value 5.4-5.5), having apparent molecular weight and pI values close to those of the legumin α-subunit. The native molecular weight of group 3 late embryogenesis abundant protein was measured as 494 ± 2 kD (average ± standard deviation; *n *= 3), suggesting a multimer of nine identical subunits. The second fraction contained the legumin polypeptide precursor, α- and β-subunits, with apparent molecular weights of 73.4, 54.8 and 21.9 kD respectively (Figure 9C; see Additional file 3D-F). Two minor bands, of 44.0 and 42.7 kD were also identified as the α-subunit (see Additional file 3G). Detection of the mass of a tryptic peptide, 423-NRGSNGIEETLCTLK-437, containing an intact cleavage site was characteristic of the precursor. A complex pattern of elution was observed after size exclusion chromatography (Figure 9B and 9C). A first peak contained all three major polypeptides, with a molecular weight of 704 ± 36 kD, suggesting possible associations between trimers of the precursor and hexamers of α- and β-subunit. A second peak contained the α-subunit alone, with a molecular weight of 282 ± 2 kD, suggesting a hexamer. Recovery of the α-subunit alone may be related to the inclusion of reducing agent during purification.

## Discussion

Profiling of free amino acids in BAT93 seeds enabled a rational selection of developmental stages to generate ESTs, which correspond to distinct phases of γ-Glu-*S*-methyl-Cys accumulation. Analysis of general GO categories related to primary metabolism, photosynthesis, response to water deficit and nutrient reservoir activity validated the representativeness of ESTs at the different stages. Free amino acid and GO category profiles confirm and extend the findings of Fait et al. [48] on the metabolic transitions to storage product accumulation and desiccation in *Arabidopsis *seeds. They highlighted a decrease in the levels of free amino acids, particularly Asn, Gln and Lys, during reserve accumulation, indicative of metabolic transformations and efficient incorporation into storage proteins. Nitrogen-rich amino acids, Asn, Lys and Arg, were transiently elevated during the transition to seed desiccation, suggesting their role as a nitrogen source to support germination prior to the mobilization of storage protein. The same was true of aromatic amino acids, Trp, Phe and Tyr, which may support the biosynthesis of shikimate-derived compounds for defense and indole-acetic acid during germination. Steady-state transcript levels of most enzymes of primary metabolism examined had reduced levels during the active phase of reserve accumulation.

The biphasic accumulation of γ-Glu-*S*-methyl-Cys is consistent with a function of *S*-methyl-Cys as a storage form of excess sulfur. During the lag phase, incorporation of sulfur amino acids into storage proteins may efficiently compete with the biosynthesis of *S*-methyl-Cys. Based on the high levels of free *S*-methyl-Cys detected at stages III and IV, substantial flux through this metabolite may lead to accumulation in the γ-glutamyl dipeptide form. The rise in Arg levels between stages III to V suggests a transient accumulation of nitrogen into this nitrogen-rich amino acid in anticipation of active storage protein accumulation. This increase may be mediated by arginosuccinate lyase activity, whose transcript is overrepresented during stage IV.

Analysis of GO categories related to sulfur metabolism revealed the occurrence of complete pathways of sulfate assimilation, *de novo *Cys and Met biosynthesis. Similar findings have been reported for soybean seed [49]. These data may be interpreted in relation with current understanding of the sulfur nutrition of legume seed. The main sources of sulfur transported to the seed are expected to be *S*-methylmethionine [50] and homoglutathione [51]. In soybean, most sulfate appears to be converted to homoglutathione in the pod, prior to uptake by developing cotyledons, although some is detected in developing seed [52]. However, under sulfur-limiting conditions, glutathione exclusively, and no sulfate, is translocated to the seed, according to a model developed in wheat [51]. Interestingly, *Arabidopsis *knock-out mutants of sulfate transporters that have been characterized so far show increased sulfate content in mature seed, suggesting a function in intracellular transport rather than uptake by the embryo [53,54]. Beside the generation of sulfide for *de novo *Cys biosynthesis, the sulfate assimilatory pathway is required for the biosynthesis of the activated nucleotide 3'-phospho-5'-adenosinephosphosulfate, the universal donor in sulfate transfer reactions. Adenylyl-sulfate kinase activity, forming 3'-phospho-5'-adenosinephosphosulfate, which competes with adenylyl-sulfate reductase for its substrate, was represented in the EST dataset (Figure 4). Cystathionine γ-synthase and -β-lyase likely provide homocysteine as an acceptor for methyl transfer from *S*-methylmethionine, catalyzed by homocysteine *S*-methyltransferase [55], while Met synthase is essential to recycle homocysteine produced in the *S*-adenosylmethionine cycle, which appeared highly active according to the high representation of the Met adenosyltransferase activity category. Further research is required to fully understand the significance of the sulfate assimilatory pathway, and the relative contributions of *de novo *Cys and Met biosynthesis in seed metabolism.

Previous results have shown that the absence of Cys-poor phaseolin and phytohemagglutinin in SMARC1N-PN1 leads to a shift of sulfur from *S*-methyl-Cys to the protein Cys pool [9]. By integrating proteomic and EST data, the deduced sequences of several proteins contributing to the increased levels of sulfur amino acids in SMARC1N-PN1 were identified. These proteins have a higher Cys than Met content, providing an explanation for the preferential increase in Cys over Met in the mutant line.

Legumin is found at relatively low levels of ca. 3% of seed protein in common bean cultivars, as compared with other grain legumes [32]. Identification of its deduced amino acid sequence establishes that it is a member of the high molecular weight 11S globulins. The mechanism leading to the evolution of high molecular weight 11S globulins involves expansion of a repeat sequence at the C-terminal end of the α-subunit. The sequences of these repeats differ between *P. vulgaris *legumin, arachin 5 and glycinin A5A4B3 (Figure 6). Legumin has four instances of the sequence NH_2_-HKEEEKEVEPLP-COOH, compared with four of the sequence NH_2_-GYDDD[E/D]RRP-COOH and eight of the sequence NH_2_-DDD[E/D]RR-COOH in arachin 5. Glycinin A5A4B3 has three repeats of the sequence NH_2_-QDEDEDEDED-COOH, compared with two in glycinin A4B3 [56]. The first instance of this sequence, spanning positions 258 to 268 of legumin, is relatively well conserved but not repeated in legumin and arachin 5. These observations suggest that expansion of repeats probably occurred, at least in part, after the separation of the lineages leading to the three species.

Purification of legumin revealed substantial accumulation of the propolypeptide in SMARC1N-PN1. A similar finding has been reported in transgenic soybean where the expression of the α-and α'-subunits of the 7S globulin α-conglycinin was suppressed [57]. Interestingly, the proglycinin was shown to be trafficked directly from the ER to the vacuole, whereas mature 11S globulins transit in the Golgi apparatus. In most legume crops, α-subunits of 11S globulins have an apparent molecular weight of approximately 40 kD. By contrast, *P. vulgaris *and *V. unguiculata *[58] share a single, dominant high molecular weight legumin whose α-subunit has an apparent molecular weight of approximately 60 kD. Similarly, in *L. japonicus*, two of three major legumins, the legumin storage proteins 2 and -3, which are closely related to the *P. vulgaris *legumin, have α-subunit apparent molecular weights of 55 and 60 kD, respectively [36]. In *G. max*, an additional cleavage site in proglycinin gives rise to A5 and A4 subunits, having a reduced molecular weight [56]. The significance of the 10 kD difference between the predicted and apparent molecular weight of the major legumin α-subunit, and the detection of minor α-subunit polypeptides of 44.0 and 42.6 kD is unclear. The minor polypeptides may represent partial degradation products, and the discrepancy in molecular weight may be due to an effect of the Glu-rich domain on electrophoretic behavior. Alternatively, these differences may arise by a possible post-translational modification of the mature α-subunit. The absence of this modification in prolegumin, whose predicted and apparent molecular weight is similar, would reflect differences in trafficking, as already noted in soybean. Among high molecular weight legumins in other legume crops, the α-subunit of *P. sativum *minor small legumin accumulates only as degradation products ranging from 21 to 32 kD, which was interpreted as an outcome of early mobilization [59], while only the β-subunit of arachin 5 was identified in the mature seed proteome of *A. hypogaea *[60].

Although phaseolin and lectins have been characterized in great detail, the number and identity of genes encoding these proteins is still unknown. In the present study, there was evidence for only two phaseolin genes in the BAT93 EST dataset. Beside the APA locus on linkage group B4, two lectin loci have been mapped on linkage group B7 [61,62]. EST, proteomic [11], cDNA [26] and genomic sequence data [62] can be integrated to provide information on the composition of the APA locus in different *P. vulgaris *genotypes. In BAT93 and *ARC5 *genotypes, a gene order consisting in *lec4-B17*/*pha-E*/phytohemagglutinin/α-amylase inhibitor-like protein can be inferred. In *ARC1*, *arc3 *and *arc4 *genotypes, the arcelin-5 phytohemagglutinin appears to be substituted with *pha-L*. *PDLEC2 *encoding leucoagglutinating phytohemagglutinin, isolated from a phytohemagglutinin-deficient Pinto cultivar [29], whose expression is elevated in SMARC1N-PN1, may be one of the lectin genes located outside of the APA locus.

## Conclusions

The BAT93 EST collection provides a foundation to initiate further studies of sulfur amino acid metabolism in developing seed, as all relevant pathways and enzyme activities are represented. The results presented here are consistent with a mechanism whereby sulfur can be efficiently partitioned between *S*-methyl-Cys and Cys, but its metabolic basis is not understood. The identification and characterization of sulfur-rich proteins whose levels are increased in the absence of phaseolin and major lectins provides an explanation for the preferential increase in Cys over Met.

## Methods

### Plant material and growth conditions

BAT93, a line representative of the Mesoamerican gene pool of common bean (*Phaseolus vulgaris*) [20], was grown as previously described [63]. Developing seed samples were harvested randomly from about twenty plants. SMARC1N-PN1 [3] was grown in the field in London, ON, in 2008.

### Extraction and quantification of free amino acids

Amino acids were extracted and quantified by HPLC after derivatization with phenylisothiocyanate as previously described [9], except that extraction was performed in ethanol:water (70:30), which is optimal for sulfur containing γ-glutamyl dipeptides [64]. Replicate samples consisted in independent pools of eight seeds which were ground in liquid nitrogen, and 100 mg of ground tissue was used for extraction. γ-Glu-*S*-methyl-Cys and γ-Glu-Leu standards were from Bachem Americas (Torrance, CA).

### RNA extraction

RNA was extracted using a modified lithium chloride precipitation method [63]. RNA was quantified by spectrophotometry on a NanoDrop 2000 (Thermo Scientific, Wilmington, DE) and its quality evaluated from A_260/280 _ratio and agarose gel electrophoresis. Poly A^+ ^RNA was isolated using the Ambion Poly(A)Purist mRNA purification kit (Applied Biosystems, Streetsville, ON). Its quality was analyzed with a 2100 Bioanalyzer (Agilent Technologies, Mississauga, ON) at the London Regional Genomics Centre, ON.

### cDNA library construction

Standard and normalized cDNA libraries were prepared for each developmental stage. cDNA was synthesized from 1 μg poly A^+ ^RNA using the SMART cDNA construction kit (Clontech Laboratories, Mountain View, CA). SMART-amplified cDNA was normalized using the Trimmer-Direct cDNA normalization kit (Evrogen, Moscow, Russia). *Sfi*I-digested cDNA was purified using the QIAquick PCR purification kit (Qiagen, Mississauga, ON). cDNA was size fractionated on a 1% agarose gel run at 45 V until the bromophenol blue dye was 2 cm away from the well, and the band was excised in two fractions corresponding to 0.5 to 1.5 kb and greater than 1.5 kb. cDNA was extracted using the QIAquick gel extraction kit (Qiagen), and the two fractions were ligated separately to a modified pBluescript II KS+ (Agilent) containing *Sfi*I cloning sites. Ligation reactions were purified using the MinElute reaction cleanup kit (Qiagen). The ligation product was transformed into ElectroMAX DH10B T1-phage resistant competent cells (Invitrogen, Burlington, ON).

### EST sequencing and analysis

Culture plates (384-well) were inoculated with a Norgren Systems CP7200 colony picker (Ronceverte, WV). Plasmids were amplified using TempliPhi (GE Healthcare Life Sciences, Baie d'Urfé, QC) and cycle sequenced using Applied Biosystems BigDye chemistry and a BioRAPTR FRD microfluidic workstation (Beckman Coulter Canada, Mississauga, ON) for amplification and sequencing reaction set-up. Completed 384-well sequencing plates were processed with Applied Biosystems 3730xl DNA analyzers. Most clones were sequenced from the 5' end using M13 Reverse primer; a small number were also sequenced with the M13-20 primer. EST processing involved quality-trimming, vector-masking, low-complexity masking, and poly-A trimming using custom perl scripts. Assembly was performed with TGICL [65] and the results stored in the FIESTA-2 database at the Plant Biotechnology Institute of the National Research Council of Canada. A total of 30,147 ESTs were assembled into 3,658 contigs and 6,027 singletons. Annotation was done by BLASTx against UniProt Plants version 15 where accessions with uninformative annotations such as "unknown protein", "putative predicted protein", "shotgun sequence from scaffold", etc. had been removed (June 19, 2009) [66]. GO annotations were transferred from the best BLASTx hit to TAIR version 8 (June 8, 2009) if the e value was smaller than or equal to 1^-10^. Contigs and singletons reported in Figure 5 were verified for the absence of cloning or sequencing artifacts.

### RACE and cDNA cloning

Total RNA from SMARC1N-PN1 (20 mg seed weight) was digested with amplification grade DNase I (Invitrogen). First strand cDNA was synthesized using ThermoScript RT-PCR system from 1 μg RNA (Invitrogen). 5' RNA ligase mediated RACE was performed with Ambion FirstChoice RLM-RACE Kit (Applied Biosystems) using *Taq *DNA polymerase (New England Biolabs, Pickering, ON). 5' RACE gene specific outer (O) and inner (I) primers were the following: for legumin, Leg-O, 5' -TCCGCCACCATAAAGTTCTGT-3' and Leg-I, 5'-CTAACTTGTCCTTGCCCCTCG-3'; and for albumin-2, Alb2-O, 5'-TTACATCAGGAAAGGAATCAGGC-3' and Alb2-I, 5'-TGTTTTGTGACGAGGATAAGGTG-3'. RACE products were either blunted with the Klenow fragment of DNA polymerase I and cloned into the Zero Blunt TOPO vector (Invitrogen) or cloned directly into the pGEM-T Easy vector (Promega, Madison, WI). Full-length cDNAs were cloned by RT-PCR with Pfx50 DNA polymerase (Invitrogen) into the Zero Blunt TOPO vector using the following primers: for legumin, Legumin-F, 5'-ACCAACCCATTCACCACTTC-3' and Legumin-R, 5'-AAGAAAGGCTTGCTAGGATGG-3'; and for albumin-2, Albumin2-F, 5'-AAGCATCCTCAAATCAAATCA-3' and Albumin2-R, 5'-AACCAAACCACCCACTTTTA-3'. Multiple sequence alignment and phylogenetic analysis with PHYLIP were performed as previously described [63]. Triticin was used as outgroup.

### Protein extraction and purification

Soluble protein was extracted from mature seed according to VandenBosch et al. [67], except that 50 mM Tris-HCl, pH 7.5 was replaced with 50 mM Tris-HCl, pH 8.0. Protein concentration was measured with the Bio-Rad Protein Assay solution and bovine serum albumin as standard. Protein precipitated with saturated ammonium sulfate between 50 to 60% (w/v) was dissolved in 50 mM bis-Tris-HCl, pH 6.5 containing 14 mM 2-mercaptoethanol and desalted on a PD-10 column (GE Healthcare Life Sciences). The extract was separated by ion exchange chromatography on a HiPrep 16/10 Q FF column, using an ÄKTApurifier system (GE Healthcare Life Sciences). Protein was eluted with a linear gradient of 0 to 0.5 M NaCl. Purified fractions of interest were concentrated using an Amicon Ultra-15 centrifugal filter unit with Ultracel-10 membrane (Millipore, Billerica, MA) prior to size exclusion chromatography on a HiLoad Superdex 75 prep grade column (GE Healthcare Life Sciences) using 50 mM bis-Tris-HCl, 150 mM NaCl containing 14 mM 2-mercaptoethanol as buffer. Proteins were further chromatographed by size exclusion on a Superose 6 10/300 GL column. Molecular weight was determined from a plot of the partition coefficient, K_av_, versus the logarithm of the molecular weight of protein standards. The following proteins were used for calibration: thyroglobulin (669 kD); ferritin (440 kD); and catalase (232 kD).

### Protein identification

To confirm protein and cDNA identity, LC-MS/MS data from 2-D gel spots [11] was used for *de novo *sequencing with PEAKS Studio v. 4.5 (Bioinformatics Solutions Inc., Waterloo, ON) [68] as previously described, and compared to conceptual cDNA translations. Protein fractions eluted from the HiLoad Superdex 75 prep grade column, and 44.0 and 42.7 kD legumin α-subunit present in Superose 6 10/300 GL fractions, were separated by SDS-PAGE on 12% polyacrylamide gels. Bands were excised, digested with trypsin and the resulting peptides were subjected to LC-MS for protein identification. For group 3 late embryogenesis abundant protein, and 44.0 and 42.7 kD legumin α-subunits, LC-MS/MS was performed as previously described for 2-D gel spots [11], except that the gradient was lengthened to 90 min. The 44.0 and 42.7 kD legumin α-subunits were identified by Mascot search of the assembled EST database, to which the legumin cDNA sequence had been appended, using an in-house server [69]. For the major legumin bands, peptides were analyzed by LC-MS using an Alliance 2690 HPLC and a model LCT orthogonal acceleration time-of-flight mass spectrometer (Waters, Mississauga, ON). Samples were diluted to 100 μL with 100:0.1 (v/v) water-formic acid. A 50 μL portion was injected into a 100 μL loop attached to a 10 port valve (VICI Valco Canada, Brockville, ON) and the valve was switched to permit transfer to a 1 mm ID × 5 mm C18 PepMap 100 trapping column (Dionex, Bannockburn, IL), using 100:0.1 (v/v) water-formic acid flowing at 0.1 mL/min from an auxiliary high pressure pump. After 10 min transfer/washing, the valve was switched to place the trapping column in line with a 1 mm ID × 150 mm C18 PepMap 100 analytical column (Dionex) and the peptides were eluted with a gradient of water-acetonitrile-formic acid flowing at 30 μL/min. An ACURATE flow splitter (Dionex) was used to reduce the flow of 0.3 mL/min from the HPLC to this level. Solvent A was 90:10:0.1 (v/v/v) water-acetonitrile-formic acid and solvent B was 10:90:0.1 water-acetonitrile-formic acid. The gradient conditions were 5 min at 100% A, a linear increase to 40% B from 5 to 35 min, a 5 min hold at 40% B followed by a return to 100% A at 45 min and a 15 min equilibration time. The column effluent was transferred to a Megaflow electrospray probe of the mass spectrometer where the components were ionized and subsequently analyzed. The ion source was operated in positive mode with a capillary voltage of 3 kV with nitrogen as desolvation gas flowing at 250 L/h and heated to 250°C. Mass spectra were acquired from 85 to 1500 m/z. The cone voltage was switched between 20 and 50 V at 1.1 sec intervals during data acquisition to permit fragmentation of individual peptides under favorable conditions of chromatographic separation and high peptide concentration. The LC-MS system was operated with MassLynx v. 4.0 and calibrated using a mixture of horse heart myoglobin and trypsinogen. Peak lists were generated from MassLynx raw data files using Mascot Distiller v. 2.2.1 (Matrix Science, Boston, MA). Peptide fragment data for each sample was processed by examining chromatographic peaks for the purity of their 20 V spectra, combining 50 V spectra across a suitable peak as representative of fragments of a single peptide and saving the data as a mass vs. intensity text file. These text files for the peptides in a sample were manually combined into a text file in .pkl format representative of the sample. Peptide mass fingerprint data was analyzed against the conceptual translation using PAWS (Digilab, Holliston, MA) and peptide sequence tags derived with Peaks Studio.

### Statistical analysis

*K*-means and hierarchical clustering was performed with GeneSpring GX v. 11.5 (Agilent).

### Accession numbers

Nucleotide sequence data from this article has been deposited in the GenBank database under accession numbers [GenBank ID: GW884178 to GW914324] for seed ESTs; [GenBank ID: HM240256] for legumin; [GenBank ID: HM240257] for albumin-2; [GenBank ID: HM240258] for defensin D1; [GenBank ID: HM240259] for defensin D2; [GenBank ID: HM240260] for albumin-1A; [GenBank ID: HM240261] for albumin-1B; [GenBank ID: HM240262] for albumin-1D; [GenBank ID: HM240263] for albumin-1F; [GenBank ID: HM240264] for albumin-1E; [GenBank ID: HM240265] for albumin-1C; and [GenBank ID: HM240266] for albumin-1G. Additional amino acid sequence data can be found under accession numbers [UniProt ID: Q41115] for α-phaseolin; [UniProt ID: Q43632] for phaseolin encoded by *Phs*; [UniProt ID: Q8RVX5] for lectin encoded by *lec4-B17*; [UniProt ID: Q8RVX6] for phytohemagglutinin encoded by *pha-E*; [UniProt ID: P15231] for leucoagglutinating phytohemagglutinin encoded by *PDLEC2*; [UniProt ID: Q43628] for phytohemagglutinin; [UniProt ID: Q6J2U4] for α-amylase inhibitor-1; [UniProt ID: Q9SMH0] for α-amylase inhibitor-like protein; [UniProt ID: Q9SB11] for glycinin A5A4B3 (*G. max*); [UniProt ID: Q647H1] for arachin 5; [UniProt ID: B5U8K1] for legumin storage protein 2; [UniProt ID: B5U8K2] for legumin storage protein 3; [UniProt ID: Q39921] for glycinin A5A4B3 (*G. soja*); [UniProt ID: A3KEY8] for glycinin A3B4 (*G. soja*); [UniProt ID: P04347] for glycinin A3B4 (*G. max*); [UniProt ID: Q3HW60] for glycinin G7; [UniProt ID: O24294] for minor small legumin; [UniProt ID: Q43673] for legumin-related high molecular weight polypeptide; [UniProt ID: P05190] for legumin B (*V. faba*); [UniProt ID: Q41703] for legumin B (*V. sativa*); [UniProt ID: P05692] for legumin J; [UniProt ID: P11828] for glycinin G3; [UniProt ID: P04776] for glycinin G1; [UniProt ID: P04405] for glycinin G2; [UniProt ID: Q0GM57] for iso-Arah3; [UniProt ID: Q5I6T2] for arachin Ahy-4; [UniProt ID: Q647H2] for arachin Ahy-3; [UniProt ID: B5U8K6] for legumin storage protein 5; [UniProt ID: Q9T0P5] for legumin A (*P. sativum*); [UniProt ID: Q41702] for legumin A (*V. sativa*); [UniProt ID: Q99304] for legumin A2; [UniProt ID: Q9SMJ4] for legumin (*C. arietinum*); [UniProt ID: Q53I54] for legumin-like protein; [UniProt ID: B2CGM6] for triticin; [UniProt ID: Q43680] for seed albumin (*V. radiata*); [UniProt ID: P08688] for albumin-2 (*P. sativum*); [UniProt ID: Q8W434] for defensin D2 (*V. radiata*); [UniProt ID: C1K3M7] for defensin (*V. unguiculata*); [UniProt ID: Q7XZC2] for albumin-1 (*P. vulgaris*); [UniProt ID: Q39837] for albumin-1 (*G. max*).

## Authors' contributions

FY performed most experiments. AP constructed cDNA libraries. RC performed LC-MS analyses. AS developed protocols for, and provided the high throughput Sanger DNA sequencing. SH co-supervised FY and helped draft the manuscript. FM designed the study and wrote the manuscript. All authors read and approved the final manuscript.

## Supplementary Material

Additional file 1 **Free amino acid profiles in developing seeds of BAT93**. Click here for file

Additional file 2 **Percent of ESTs assigned to a Gene Ontology category per developmental stage (%)**. Click here for file

Additional file 3 **Legumin (A) and albumin-2 (B) tryptic peptides identified by de novo sequencing of LC-MS/MS data; Identification of purified proteins after size exclusion chromatography: group 3 late embryogenesis abundant protein (C); legumin precursor (D), α- -54.8 kD (E), β- (F), and α- -44.0 and 42.7 kD (G) subunits**. Click here for file
