# Supplementary material for: Analysis of common bean expressed sequence tags identifies sulfur metabolic pathways active in seed and sulfur-rich proteins highly expressed in the absence of phaseolin and major lectins
Source: BMC Genomics. 2011 May 26;12:268. doi: 10.1186/1471-2164-12-268 (PMC3115882; doi:10.1186/1471-2164-12-268)
Supplement: Additional file 1 — Free amino acid profiles in developing seeds of BAT93. [file 1471-2164-12-268-S1.PDF]

Free amino acid profiles in developing seeds of BAT93

Day after fertilization/ seed weight (mg)/developmental stage	9/8 III – heart	13/16 IV – cotyledon	15/36 IV – cotyledon	18/98 V – cotyledon	22/180 VI – maturation	27/288 VII – maturation	33/489 VIII – maturation	Mature/ 163
Asp	1.2 ± 0.3	1.2 ± 0.1	0.82 ± 0.05	0.55 ± 0.02	0.62 ± 0.04	0.79 ± 0.13	1.16 ± 0.13	0.71 ± 0.07
Glu	3.0 ± 0.6	3.0 ± 0.2	3.1 ± 0.2	4.0 ± 0.1	1.6 ± 0.1	1.7 ± 0.3	0.91 ± 0.08	0.68 ± 0.07
Asn	3.9 ± 0.6	3.4 ± 1.2	2.3 ± 0.1	2.3 ± 0.1	0.34 ± 0.02	0.12 ± 0.01	0.32 ± 0.04	n. d.
Ser	1.3 ± 0.2	1.2 ± 0.1	0.79 ± 0.03	0.53 ± 0.02	0.21 ± 0.01	0.21 ± 0.03	0.18 ± 0.02	0.058 ± 0.017
Gln	21 ± 5	15 ± 4	13 ± 1	7.7 ± 0.2	0.49 ± 0.03	0.21 ± 0.03	0.13 ± 0.02	n. d.
Gly	0.32 ± 0.05	0.24 ± 0.03	0.19 ± 0.01	0.17 ± 0.01	0.11 ± 0.01	0.10 ± 0.01	0.057 ± 0.012	0.12 ± 0.02
His	0.80 ± 0.22	0.88 ± 0.03	0.64 ± 0.03	0.60 ± 0.02	0.43 ± 0.02	0.083 ± 0.016	0.30 ± 0.04	0.95 ± 0.08
γ-Glu-*S*-methyl-Cys	0.050 ± 0.010	0.15 ± 0.06	0.84 ± 0.05	2.4 ± 0.1	2.3 ± 0.1	1.8 ± 0.4	3.2 ± 0.5	9.4 ± 1.0
Arg	3.4 ± 0.8	3.6 ± 0.6	4.2 ± 0.3	6.0 ± 0.1	0.87 ± 0.05	0.24 ± 0.04	0.57 ± 0.08	4.0 ± 0.4
Thr	0.72 ± 0.14	0.75 ± 0.05	0.62 ± 0.04	0.51 ± 0.03	0.30 ± 0.02	0.19 ± 0.02	0.27 ± 0.04	0.12 ± 0.01
Ala	5.8 ± 1.9	3.5 ± 1.2	1.3 ± 0.1	1.0 ± 0.1	0.33 ± 0.01	0.20 ± 0.01	0.37 ± 0.05	0.50 ± 0.06
Pro	0.34 ± 0.04	0.31 ± 0.07	0.23 ± 0.01	0.17 ± 0.01	0.080 ± 0.004	0.066 ± 0.005	0.20 ± 0.01	0.24 ± 0.11
γ-Glu-Leu	0.090 ± 0.015	0.092 ± 0.009	0.092 ± 0.004	0.31 ± 0.02	0.67 ± 0.02	0.73 ± 0.10	0.74 ± 0.08	1.8 ± 0.2
Tyr	0.041 ± 0.009	0.077 ± 0.040	0.069 ± 0.007	0.067 ± 0.015	0.048 ± 0.002	0.035 ± 0.008	0.027 ± 0.003	0.051 ± 0.006
S-methyl-Cys	0.41 ± 0.15	0.40 ± 0.05	0.41 ± 0.02	0.21 ± 0.02	0.095 ± 0.001	0.067 ± 0.019	0.081 ± 0.006	0.16 ± 0.02
Val	0.55 ± 0.24	0.84 ± 0.04	0.62 ± 0.03	0.56 ± 0.02	0.23 ± 0.01	0.17 ± 0.02	0.29 ± 0.04	0.20 ± 0.02
Met	1.8 ± 0.3	1.6 ± 0.3	1.6 ± 0.1	0.87 ± 0.04	0.13 ± 0.01	0.10 ± 0.02	0.040 ± 0.006	0.044 ± 0.005
Ile	0.16 ± 0.02	0.41 ± 0.35	0.18 ± 0.01	0.18 ± 0.01	0.16 ± 0.01	0.11 ± 0.01	0.25 ± 0.03	0.14 ± 0.01
Leu	0.28 ± 0.05	0.44 ± 0.01	1.4 ± 0.1	1.2 ± 0.03	0.13 ± 0.01	0.083 ± 0.009	0.15 ± 0.03	0.12
Phe	0.10 ± 0.01	0.16 ± 0.01	0.12 ± 0.01	0.13 ± 0.01	0.086 ± 0.038	0.052 ± 0.014	0.22 ± 0.11	0.73 ± 0.02
Lys	0.062 ± 0.017	0.093 ± 0.016	0.17 ± 0.01	0.30 ± 0.02	0.046 ± 0.001	0.030 ± 0.002	0.018 ± 0.004	0.40 ± 0.03

In nmol per mg seed weight; average ± standard deviation; *n* = 3; n. d.: not determined
