# Supplementary material for: Analysis of common bean expressed sequence tags identifies sulfur metabolic pathways active in seed and sulfur-rich proteins highly expressed in the absence of phaseolin and major lectins
Source: BMC Genomics. 2011 May 26;12:268. doi: 10.1186/1471-2164-12-268 (PMC3115882; doi:10.1186/1471-2164-12-268)
Supplement: Additional file 2 — Percent of ESTs assigned to a Gene Ontology category per developmental stage (%). [file 1471-2164-12-268-S2.PDF]

Percent of ESTs assigned to a gene ontology category per developmental stage (%)

GO category and number/Developmental stage	IV	V	VI	VII
embryonic development ending in seed dormancy 0009793	1.66	0.89	0.55	1.45
response to gibberellin stimulus 0009739	1.46	0.44	0.27	0.33
hormone-mediated signaling 0009755	0.66	0.34	0.34	0.41
response to auxin stimulus 0009733	0.47	0.15	0.08	0.30
response to abscisic acid stimulus 0009737	0.26	0.17	0.25	0.21
gibberellic acid mediated signaling 0009740	0.30	0.19	0.23	0.12
auxin mediated signaling pathway 0009734	0.19	0.03	0.02	0.11
response to hormone stimulus 0009725	2.18	0.84	0.59	1.01
response to jasmonic acid stimulus 0009753	0.25	0.19	0.11	0.14
response to salicylic acid stimulus 0009751	0.11	0.08	0.15	0.23
response to abiotic stimulus 0009628	2.13	1.26	1.33	1.75
response to temperature stimulus 0009266	1.36	1.26	1.33	1.75
response to radiation 0009314	0.46	0.48	0.32	0.20
response to water 0009415	0.22	0.09	0.21	0.32
response to stress 0006950	2.99	1.26	1.33	1.75
response to oxidative stress 0006979	0.92	0.95	0.53	0.51
response to cold 0009409	0.78	0.38	0.44	0.53
response to heat 0009408	0.6	0.89	0.91	1.25
response to osmotic stress 0006970	0.33	0.15	0.08	0.21
response to wounding 0009611	0.33	0.48	0.27	0.36
response to water deprivation 0009414	0.22	0.09	0.21	0.32
response to hypoxia 0001666	0.11	0.12	0.02	0.12
multicellular organismal developmental process 0044236	2.29	1.55	0.55	1.60
seed development 0048316	1.67	0.89	0.57	1.51
embryonic development 0009790	1.66	0.9	0.59	1.45
post-embryonic development 0009791	0.46	0.21	0.11	0.21
system development 0048731	0.21	0.23	0.17	0.26
metabolic process 0008152	32.27	19.81	7.62	16.43
cellular metabolic process 0044237	28.37	15.22	10.97	17.35
primary metabolic process 0044238	27.02	14.69	10.65	17.15
macromolecule metabolic process 0043170	23.07	11.83	8.96	14.54
biosynthetic process 0009058	16.29	7.36	5.14	6.97
catabolic process 0009056	2.88	1.7	1.37	2.11
generation of precursor metabolites and energy 0006091	2.35	1.61	0.97	2.05
nitrogen compound metabolic process 0006807	1.8	1.1	0.65	0.89
regulation of metabolic process 0019222	1.56	0.91	0.42	1.36
photosynthesis 0015979	0.92	0.61	0.30	0.30
secondary metabolic process 0019748	0.65	0.42	0.17	0.27
establishment of localization 0051234	4.88	3.32	2.45	3.94
macromolecule localization 0033036	1.71	0.94	0.53	1.24
transport 0006810	4.85	3.29	2.39	3.91
establishment of protein localization 0045184	1.71	0.94	0.53	1.24
establishment of cellular localization 0051649	1.54	1.02	0.66	1.15
secretion 0046903	0.29	0.24	0.17	0.20
catalytic activity 0003824	16.58	13.92	8.71	16.69
binding 0005488	14.7	9.25	6.39	11.38
structural molecule activity 0005198	12.17	4.14	3.24	4.00
transporter activity 0005215	3.44	2.35	1.82	2.53
transcription regulator activity 0030528	1.73	0.78	0.55	1.54
translation regulator activity 0045182	0.97	0.73	0.44	0.75
antioxidant activity 0016209	0.86	0.72	0.32	0.45
enzyme regulator activity 0030234	0.34	0.36	0.36	0.59
molecular transducer activity 0060089	0.23	0.18	0.04	0.18
nutrient reservoir activity 0045735	0.09	1.09	1.39	0.42
cellular protein metabolic process 0044267	18.3	8.89	6.79	10.29
sulfur compound metabolic process 0006790	0.44	0.41	0.15	0.50
amino acid metabolic process 0006250	1.67	0.93	0.61	0.77
amino acid biosynthetic process 0008652	1.28	0.50	0.42	0.50
hormone biosynthetic process 0042446	0.40	0.13	0.02	0.02
